# Künstliche Intelligenz in der Hals-Nasen-Ohren-Heilkunde

**DOI:** 10.1007/s00106-021-01095-0

**Published:** 2021-08-10

**Authors:** Stefan P. Haider, Kariem Sharaf, Philipp Baumeister, Christoph A. Reichel

**Affiliations:** 1 Klinik und Poliklinik für Hals-Nasen-Ohrenheilkunde, LMU-Klinikum, Marchioninistr. 15, 81377 München, Deutschland; 2 Department of Radiology and Biomedical Imaging, Section of Neuroradiology, Yale School of Medicine, 789 Howard Ave, 06519 New Haven, CT USA

**Keywords:** Robotik, „Machine-Learning“, Mehrschichtiges Lernen, Big Data, Kopf und Hals, Robotics, Machine learning, Deep learning, Big data, Head and neck

## Abstract

**Hintergrund:**

Die fortschreitende Digitalisierung ermöglicht zunehmend den Einsatz von künstlicher Intelligenz (KI). Sie wird Gesellschaft und Medizin in den nächsten Jahren maßgeblich beeinflussen.

**Ziel der Arbeit:**

Darstellung des gegenwärtigen Einsatzspektrums von KI in der Hals-Nasen-Ohren-Heilkunde und Skizzierung zukünftiger Entwicklungen bei der Anwendung dieser Technologie.

**Material und Methoden:**

Es erfolgte die Auswertung und Diskussion wissenschaftlicher Studien und Expertenanalysen.

**Ergebnisse:**

Durch die Verwendung von KI kann der Nutzen herkömmlicher diagnostischer Werkzeuge in der Hals-Nasen-Ohren-Heilkunde gesteigert werden. Zudem kann der Einsatz dieser Technologie die chirurgische Präzision in der Kopf-Hals-Chirurgie weiter erhöhen.

**Schlussfolgerungen:**

KI besitzt ein großes Potenzial zur weiteren Verbesserung diagnostischer und therapeutischer Verfahren in der Hals-Nasen-Ohren-Heilkunde. Allerdings ist die Anwendung dieser Technologie auch mit Herausforderungen verbunden, beispielsweise im Bereich des Datenschutzes.

## Abstract (en)

**Background:**

The continued advancement of digitalization increasingly allows deployment of artificial intelligence (AI) algorithms, leveraging profound effects on society and medicine.

**Objective:**

This article aims to provide an overview of current developments and futures perspectives of AI in otorhinolaryngology.

**Materials and methods:**

Scientific studies and expert analyses were evaluated and discussed.

**Results:**

AI can increase the value of current diagnostic tools in otorhinolaryngology and enhance surgical precision in head and neck surgery.

**Conclusion:**

AI has the potential to further improve diagnostic and therapeutic procedures in otorhinolaryngology. This technology, however, is associated with challenges, for example in the domain of privacy and data security.

Künstliche Intelligenz (KI, „artificial intelligence“, AI) wird als die größte treibende Kraft in der Digitalisierung angesehen, welche mit dem Schritt von der „rechnenden“ zur „kognitiven“ Informatik die Welt noch schneller und umfassender verändern wird als die bisherige digitale Revolution: Zusätzlich zu vorprogrammierten Abläufen ermöglicht KI zur Problemlösung eine zunehmende Lernfähigkeit („Machine-Learning“, s. unten), Selbstständigkeit und Interaktivität digitaler Systeme und Maschinen. Es wird deshalb erwartet, dass diese Technologie tiefgreifenden Einfluss auf unsere Gesellschaft nimmt und damit neben großen Möglichkeiten auch ernstzunehmende Herausforderungen verbunden sind.

Aus diesem Grund hat der Deutsche Bundestag im Juni 2018 mit der Einsetzung der Enquete-Kommission „Künstliche Intelligenz – Gesellschaftliche Verantwortung und wirtschaftliche, soziale und ökologische Potenziale“ dieses zentrale Thema unserer Zeit aufgegriffen. Nach knapp zweijähriger Arbeit wurde nun der achthundert Seiten lange Abschlussbericht der Kommission veröffentlicht [8]. In intensiver Zusammenarbeit der Parlamentarier mit Sachverständigen aus Wissenschaft und Technik wurden darin verschiedenste Teilbereiche der KI erörtert und Handlungsempfehlungen ausgesprochen. In deren Zentrum steht, diese Technologie auf Wohl und Würde des Menschen auszurichten und zum gesellschaftlichen Nutzen einzusetzen. Zur Vermeidung von Risiken automatisierter Entscheidungen durch KI müssten die der Entscheidungsfindung zugrunde liegenden Prozesse jedoch transparent, nachvollziehbar und durch den Menschen überprüfbar sein. So sollte es gelingen, das enorme Potenzial von KI für unsere Gesellschaft gewinnbringend auszuschöpfen. In diesem Zusammenhang wurde an vorderster Stelle die Bedeutung von KI für die Patientenversorgung durch uns Ärzt*innen genannt. Doch welchen Stellenwert besitzt KI in unserem ärztlichen bzw. HNO-ärztlichen Handeln? Dieser Übersichtsartikel soll seinen Leser*innen einen Überblick über bisherige Entwicklungen und zukünftige Perspektiven dieser neuartigen Technologie in unserem Fachgebiet verschaffen.

## Terminologie

**Figure 1:**
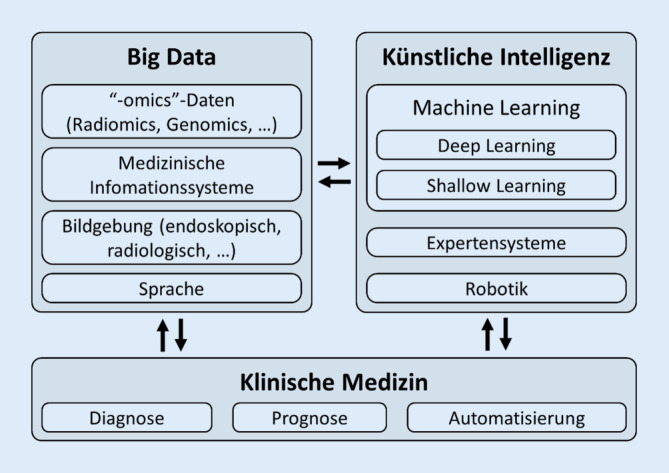

Zusammen mit dem Begriff KI sind bereits weitere Fachausdrücke Teil unseres Sprachgebrauchs geworden (Abb. 1). So bezeichnet der Terminus Big Data große Mengen an Daten verschiedenster Art und unterschiedlichsten Ursprungs. Sie zeichnen sich durch die definierende Eigenschaft aus, in Umfang und Komplexität die Möglichkeiten der manuellen Erfassung und Validierung sowie konventionellen Verarbeitung und Analyse zu übersteigen. KI-Systeme ermöglichen nicht nur die Verarbeitung enormer Datenmengen in konstanter Qualität, sondern auch die Erkennung komplexer Beziehungen und Muster in Daten, welche dem menschlichen Gehirn oder konventioneller statistischer Auswertung nicht im selben Ausmaß zugänglich wären. Ein Beispiel hierfür sind die im medizinischen Bereich täglich generierten patientenbezogenen Daten, welche durch die fortschreitende Digitalisierung – beispielsweise in medizinischen Informationssystemen – immer besser verfügbar werden. In der Hals-Nasen-Ohren-Heilkunde betrifft dies derzeit neben allgemeinen Patientendaten v. a. radiologische, audiologische und fotografische Befunde sowie Videoaufnahmen von Untersuchungen und Operationen [26].

Um diese wachsenden Datenmengen und komplexen Datenstrukturen weiter verarbeiten zu können, haben sich im medizinischen Bereich Machine-Learning(ML)-Anwendungen etabliert. Unter dem Überbegriff der KI nimmt ML eine Sonderposition ein: Während KI sämtliche Systeme der digitalen Datenverarbeitung umfasst, welche menschliche Intelligenz und Fähigkeiten nachahmen, zeichnet sich ML durch selbstständige „Lernfähigkeit“ aus: Hier kommen Computer-Algorithmen zum Einsatz, welche Muster und Gesetzmäßigkeiten in Datensätzen erkennen, somit anhand von Trainingsdaten „intelligente“ Funktionalität selbstständig erwerben und in hochdimensionalen mathematischen Modellen zur Lösung komplexer Problemstellungen vereinen können [7, 9]. Unter KI fallen dagegen auch Systeme, die „lediglich“ vorprogrammierte, von Experten festgelegte Funktionalität umsetzen, ohne die für ML charakteristische „Lernfähigkeit“. KI und ML werden in zunehmendem Maße auch in der Hals-Nasen-Ohren-Heilkunde eingesetzt (s. unten).

**Figure 2:**
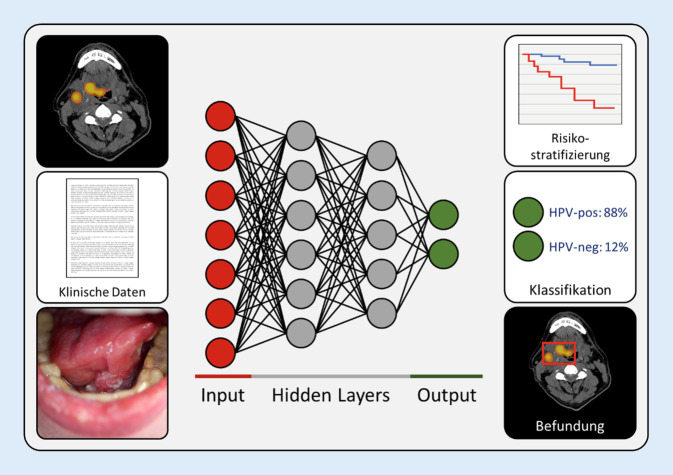

Mit dem stetigen Anstieg an verfügbarer Computerrechenleistung erlangte in den vergangenen Jahren dabei insbesondere die ML-Algorithmusklasse der sog. künstlichen neuronalen Netzwerke (KNN, „artificial neural networks“, ANN) eine herausragende Stellung. KNN sind Modelle der Informationsverarbeitung, welche in ihrer Struktur, in ihrer elementaren Vernetzung und im weitesten Sinne in ihrer Funktionalität biologischen Neuronennetzwerken nachempfunden sind (Abb. 2; [20]). Besonders hervorzuheben ist das sog. mehrschichtige oder tiefe Lernen (Deep Learning), welches eine Untergruppe von KNN bildet und sich durch eine besonders komplexe Modellarchitektur auszeichnet. Diese Architektur erlaubt es, analytisch multidimensionale Sachverhalte zu erfassen und somit nahezu jeden Hypothesenraum abzubilden. Mehrschichtiges Lernen ist rechenleistungsintensiver und unter bestimmten Umständen leistungsstärker als normale KNN [7, 8, 20]. Dennoch wird „konventionelles“ ML, welches in Abgrenzung zum Deep Learning manchmal als „Shallow Learning“ bezeichnet wird, auch heute noch häufig zur Analyse medizinischer Daten eingesetzt; nämlich insbesondere dann, wenn die Komplexität der Daten oder die Größe des Datensatzes das Training eines Deep Learning-Modells nicht erfordert bzw. erschwert. Konventionelle Algorithmen arbeiten mit vordefinierten Eigenschaften („Features“) während Deep Learning-Modelle Features erlernen können (s. Beispiel unten). Gebräuchliche „konventionelle“ ML-Algorithmen sind etwa „Random-Forest-“, „Xgboost-“ und „Support Vector Machine-Algorithmen“ [7].

Bildgebende Verfahren bieten ein besonders großes Potenzial für KI-Anwendungen. In radiologischen Untersuchungen werden deshalb zunehmend sog. Radiomics-Analysen durchgeführt, welche die herkömmliche qualitativ-visuelle Interpretation der Bildgebung durch eine möglichst umfassende Extraktion zahlreicher quantitativer Marker ergänzt [9]. Erfasst werden hierbei unter anderem Form, Intensität und Textur eines definierten Bildareals, wie z. B. eines Tumors. Mithilfe dieser sog. Radiomics-Features erfolgen dann die (ML-)Analysen. Eine Weiterentwicklung dieser konventionellen Radiomics-Analysen sind „explorative“ Radiomics-Analysen, in welchen unterschiedlichste Radiomics-Features durch KNN in den vorliegenden Bilddaten direkt „erlernt“ und anschließend ausgewertet werden [20].

Die folgenden Abschnitte unseres Artikels bieten einen Überblick zum Einsatz von KI in verschiedenen Teilbereichen der Hals-Nasen-Ohren-Heilkunde. Hierbei werden schwerpunktmäßig Arbeiten mit potenzieller Bedeutung für unsere tägliche klinische Arbeit vorgestellt. Für eine umfangreiche Auflistung sämtlicher ML- und KI-Arbeiten mit Relevanz für die Hals-Nasen-Ohren-Heilkunde sei auf die englischsprachige Übersichtsarbeit unter Referenz [29] verwiesen, welche unter anderem auch (experimentelle) Arbeiten zu KI-Analyse und -Verarbeitung von elektrischen Biosignalen (EEG, EMG) und „‑omics“-Daten (Genomics, Transcriptomics) beinhaltet [29].

## KI in der Otologie und Neurootologie

Im otologischen Bereich wurde KI bereits zur automatisierten Beurteilung radiologischer Befunde eingesetzt. In diesem Zusammenhang konnte eine Deep Learning-Analyse von 1147 Felsenbein-Computertomographien (CT) bei der Unterscheidung zwischen Normalbefund, chronischer Schleimhauteiterung und chronischer Knocheneiterung/Cholesteatom eine Korrektklassifikationsrate von 76,7 % erzielen (Sensitivität von 75 %: chron. Schleimhauteiterung, 76 %: chron. Knocheneiterung und 79 %: Normalbefund). Ähnliche Ergebnisse erbrachte die Befundung des Bildmaterials durch Radiolog*innen und HNO-Ärzt*innen [31].

In einer weiteren Studie wurde die KI-gestützte Begutachtung von Trommelfellbefunden erprobt. Dabei wurden 1366 ohrmikroskopische Aufnahmen 14 unterschiedlichen Befundkategorien (wie z. B. „Cerumen“, „Trommelfellretraktion“ oder „akute Otitis media“) mithilfe eines Deep-Learning-Algorithmus zugeordnet. Das beste Modell erzielte dabei eine Korrektklassifikationsrate von 88,7 % (Sensitivität von 86,1 % und positiver prädiktiver Wert von 90,9 % [Durchschnittswerte aller Diagnosen]), was einer präziseren Beurteilung als durch den Menschen entsprach [23].

Darüber hinaus werden derzeit ML-Modelle entwickelt, welche – basierend auf verschiedenen audiologischen Variablen – das Hörergebnis nach einem akuten Hörsturz (Remission vs. dauerhafte Hörminderung) vorhersagen sollen [3]. Im neurootologischen Bereich wurden KI-Systeme zur Nystagmus-Detektion bei der Lagerungsprobe eingesetzt. Dies erlaubte beispielsweise beim benignen paroxysmalen Lagerungsschwindel eine Deep-Learning-gestützte Identifikation des betroffenen Bogengangs [22].

Eine große Herausforderung bei der Entwicklung und Anpassung von Hörgeräten ist die suffiziente Verbesserung des Sprachverstehens in Hörumgebungen mit Störgeräuschen. Ein neuartiger Deep-Learning-basierter Sprachfilter wurde an 640.000 Stimuli trainiert und anhand zehn Normalhörender und zehn Personen mit sensorineuralem Hörverlust getestet. Durch diese Technik konnte bei beiden Gruppen eine signifikante Verbesserung des Sprachverstehens in störgeräuschbehafteten Testsequenzen nachgewiesen werden [4]. Insbesondere in neuen, unbekannten Hörumgebungen erscheint die Deep-Learning-Technologie gegenüber herkömmlichen Störgeräusch-Filtern vorteilhaft.

## KI in der Kopf-Hals-Onkologie

Die ersten Studien zu KI in der Kopf-Hals-Onkologie beschäftigten sich zunächst mit dem Einsatz von Radiomics zur radiologischen Bestimmung histopathologischer Eigenschaften von Tumoren des oberen Luft-Speise-Wegs. Unter Beteiligung der Autoren dieses Artikels wurden in diesem Zusammenhang ML-Modelle zur Einschätzung der Vergesellschaftung von oropharyngealen Plattenepithelkarzinomen mit einer Humanes-Papillomavirus(HPV)-Infektion entwickelt [10]. Diese Radiomics-Analysen umfassten fünf Fluordesoxyglukose(FDG)-Positronenemissionstomographie(PET)-CT-Datensätze von Patient*innen der Yale School of Medicine (New Haven, USA) sowie weiterer Institutionen in Kanada und in den USA, bei welchen insgesamt 2248 Bildareale von 435 Primärtumoren und 741 metastatischen Lymphknoten untersucht wurden. Nach Extraktion von jeweils 1037 PET- und CT-Radiomics-Markern wurden durch Kombination verschiedener ML-Algorithmen 360 Kandidatenmodelle generiert. Der beste Ansatz vereinte dabei mithilfe eines Xgboost-Algorithmus PET- und CT-Primärtumor-Radiomics-Features und erreichte AUC-Werte („area under the receiver operating characteristic curve“; 0,5 entspricht einer zufälligen und 1,0 einer perfekten Klassifikation) von 0,78 im Trainingsdatensatz und 0,77 im unabhängigen Testdatensatz (Sensitivität von 73 % und positiver prädiktiver Wert von 88 % im Testdatensatz). Folglich könnten derartige KI-Strategien das Potenzial zur nichtinvasiven Bestimmung der HPV-Assoziation oropharyngealer Plattenepithelkarzinome besitzen. Auf ähnliche Art und Weise wurde gezeigt, dass auch Magnetresonanztomographie (MRT)-Radiomics- [28] und DWI-MRT-Marker [24] als HPV-Assoziations-Prädiktoren fungieren können. Des Weiteren konnte die Arbeitsgruppe um Chen [5] einen Zusammenhang zwischen PET-Radiomics-Features und der Expression des Immuncheckpoint-Moleküls „programmed death-ligand 1“ (PD-L1) in Kopf-Hals-Plattenepithelkarzinomen nachweisen. Schließlich deuten Ergebnisse einer explorativen Radiomics-Analyse von Dual-Energy-CT-Untersuchungen darauf hin, dass solche KI-Algorithmen (auf Basis von Random-Forest-ML-Modellen) sogar eine präoperative Einschätzung der histopathologischen Entität von Ohrspeicheldrüsentumoren zulassen könnten [1].

Neben radiologischen Diagnoseverfahren erzeugen auch endoskopische Untersuchungen (dynamische) Bilddaten, welche durch KI-Systeme (sogar in Echtzeit) ausgewertet werden können. In einer aktuellen Studie wurden 19.000 Weißlicht-Endoskopiebilder von Stimmlippenläsionen durch ein Expertengremium aus HNO-Ärzt*innen sowie mithilfe eines Deep-Learning-Algorithmus analysiert und anschließend mit dem histopathologischen Untersuchungsergebnis (benigne Läsion vs. Leukoplakie vs. maligne Neoplasie) verglichen. Dabei schnitt das KI-System kategorieübergreifend signifikant besser ab als der Mensch (Korrektklassifikationsrate von 94 vs. 62 %) [25].

Das Vorliegen eines kapselüberschreitenden Tumorwachstums bei Lymphknotenmetastasen („extranodal extension“, ENE) kann radiologisch oftmals nicht mit ausreichender Sicherheit diagnostiziert werden. Mithilfe explorativer Deep-Learning-basierter Radiomics-Analysen wurden 655 zervikale Lymphknoten eines CT-Datensatzes in drei verschiedene Kategorien (benigne, metastatisch ENE-positiv, metastatisch ENE-negativ) klassifiziert [16]. Eine Validierungsstudie [17] erzielte unter Benutzung dieses KI-Algorithmus AUC-Werte von bis zu 0,90 (Sensitivität von 0,82, Spezifität von 0,91) für die ENE-Detektion. Bei der Detektion von Metastasen wurde eine AUC von 0,91 erreicht (Sensitivität von 0,84, positiver prädiktiver Wert von 0,88) [16]. Die computergestützte Analyse der CT-Befunde war damit präziser als die Auswertungen durch den Menschen [17]. Ähnliche Ergebnisse konnten in Radiomics-Texturanalysen von Dual-Energy-CT-Untersuchungen hinsichtlich der Differenzierung von Normalbefunden, Plattenepithelkarzinommetastasen, Lymphombefall oder entzündlicher Veränderung zervikaler Lymphknoten erzielt werden [27].

Mit Abstand am meisten Beachtung fanden bisher KI-Anwendungen aus dem Bereich der Kopf-Hals-Onkologie, welche sich mit der Prognostik des Krankheitsverlaufs bzw. der Prädiktion des Therapieansprechens beschäftigen. Als Datengrundlage dienen bei diesen Studien neben klinischen Befunden v. a. radiologische Untersuchungsergebnisse (CT, MRT oder PET-CT) [9, 18]. Die Ergebnisse dieser Arbeiten deuten darauf hin, dass die zusätzliche Verwendung von KI der gegenwärtigen diagnostischen Aufarbeitung oro-, naso- und hypopharyngealer sowie oraler und laryngealer Karzinome hinsichtlich Prognostik bzw. Prädiktion der jeweiligen Studienendpunkte überlegen ist [11, 12, 18]. Im Speziellen belegen kürzlich veröffentlichte Untersuchungen der Autoren dieses Artikels einen additiven Nutzen im Hinzuziehen von PET-CT-Radiomics-Markern im Vergleich zur alleinigen Zuhilfenahme der UICC-Staging-Klassifikation bezüglich der Prognostik von Gesamtüberleben, progressionsfreiem Überleben [12] und lokoregionärer Tumorprogression [11] bei Patient*innen mit HPV-assoziierten und nicht-HPV-assoziierten Oropharynxkarzinomen. Da das UICC-Staging auf ärztlicher Einschätzung beruht, ist für solche und ähnlich KI-Systeme „menschlicher Input“ notwendig („human in the loop AI“).

Eine häufig bei der Behandlung von Kopf-Hals-Karzinomen auftretende Nebenwirkung ist die strahlentherapieinduzierte Schädigung der Kopfspeicheldrüsen. Auf Grundlage prätherapeutischer Radiomics-Marker von Ohr- und Unterkieferspeicheldrüsen in der CT-Bildgebung lässt sich nun auch eine Vorhersage zur Ausprägung der posttherapeutischen Xerostomie treffen [30]. Mithilfe derartiger KI-Applikationen könnte folglich eine weitere Individualisierung radiotherapeutischer Maßnahmen möglich werden.

## KI in der Rhinologie

Auch für den Bereich Nase und Nasennebenhöhlen (NNH) befinden sich radiologisch-diagnostische KI-Applikationen in der Entwicklung. In ersten Studien wurden Deep-Learning-Algorithmen zunächst zur Diagnostik der Sinusitis maxillaris anhand konventioneller Röntgenaufnahmen in okzipitomentaler Projektion erprobt. Die Leistungsfähigkeit von KI wurde dabei mit der Befundung der Untersuchungen durch fünf Radiolog*innen verglichen [19]. Dabei erzielte der Deep-Learning-Algorithmus mit AUC-Werten von 0,88–0,93 (Sensitivität von 0,56–0,77, Spezifität von 0,94–0,99) eine leichtgradig signifikant bessere diagnostische Genauigkeit als die menschlichen Expert*innen mit AUC-Werten von 0,83–0,89 (Sensitivität von 0,52–0,82, Spezifität von 0,80–0,99).

Eine weitere Studie konnte zeigen, dass Deep-Learning-Algorithmen Okklusionen des osteomeatalen Komplexes bei Patient*innen mit chronischer Rhinosinusitis auf koronaren NNH-CT-Schnitten detektieren können [6]. Zudem wurde in einer anderen Arbeit ein Deep-Learning-Modell zur vollautomatisierten, volumetrischen Quantifikation von NNH-Verschattungen in NNH-CT als Surrogatparameter für das Vorliegen bzw. die Aktivität der chronischen Rhinosinusitis entwickelt [15]. Derartige Algorithmen könnten in Zukunft eine präzisere und objektivere diagnostische Erfassung und Verlaufsbeurteilung von NNH-Pathologien ermöglichen. Eine automatisierte Befundung bzw. Prädiktion des Therapieansprechens erscheint in Anlehnung an die bereits vorgestellten Arbeiten in der Kopf-Hals-Onkologie als der nächste Schritt in der Weiterentwicklung dieser Technologie.

## KI in der Kopf-Hals-Chirurgie

Neben ihrer Anwendung in Diagnostik und Prognosestellung bei Erkrankungen des Kopf-Hals-Bereichs wird KI auch im Rahmen therapeutischer Maßnahmen eingesetzt. So werden Deep-Learning-Ansätze in Studien bereits für die Operationsvorbereitung genutzt: Anhand von Fotomaterial kann beispielsweise eine KI-gestützte Planung von chirurgischen Inzisionen beim Verschluss von Lippen-Kiefer-Gaumen-Spalten durchgeführt werden, welche eine Optimierung des funktionellen und kosmetischen Ergebnisses erbringen soll [21]. Besonders vielversprechend scheint darüber hinaus der Einsatz KI-gestützter intraoperativer Bildgebungsverfahren zu sein, welche die visuelle und taktile bzw. haptische Orientierung des Chirurgen im Operationsfeld im Sinne einer erweiterten Realität (Augmented Reality, AR) ergänzen. Auf diese Weise kann für den Operateur insbesondere die Unterscheidung von erkranktem und gesundem Gewebe im Sinne einer Image-guided Surgery erleichtert werden [2].

In diesem Zusammenhang könnte sich v. a. der Einsatz von Operationsrobotersystemen als besonders nützlich erweisen: Hier liefern Kamerasysteme hochauflösendes Bildmaterial, welches zusammen mit radiologischen Daten eine mehrdimensionale Kartierung des Operationsfelds ermöglicht. Die zusätzliche Integration von Messdaten robotischer Sensoren und Aktuatoren könnte dabei sogar eine vom Operateur kontrollierte (Semi‑)Automatisierung einzelner Operationsschritte erlauben. Darüber hinaus könnte beispielsweise die Implementierung KI-gestützter optischer Verfahren wie Immunfluoreszenzmikroskopie, optische Kohärenztomographie oder Hyperspektralmikroskopie in solche „intelligenten Operationssysteme“ Tumorgrenzen noch präziser beurteilen lassen, wodurch onkologische Sicherheit und Funktionserhalt bei der chirurgischen Behandlung von Kopf-Hals-Malignomen weiter gesteigert würden [13].

## Ausblick

KI wird die Medizin in den nächsten Jahren durch eine Beschleunigung der Digitalisierung weiter verändern. In der Hals-Nasen-Ohren-Heilkunde wird diese neuartige Technologie zunehmend im Bereich der Diagnostik eingesetzt. Dabei könnte die Anwendung von KI in naher Zukunft insbesondere bei den bildgebenden Verfahren eine Verbesserung der diagnostischen Sicherheit ermöglichen und sogar eine noch frühzeitigere Erkennung und artdiagnostische Einordnung von pathologischen Veränderungen erlauben. Darüber hinaus könnte der Einsatz von KI gegenüber der herkömmlichen Auswertung bildgebender Untersuchungen einen erheblichen Mehrwert erbringen, beispielsweise bei der Prognose des Krankheitsverlaufs und bei der Vorhersage des Therapieansprechens. Auf Grundlage derartiger KI-gestützter Modelle könnten im Sinne der personalisierten Medizin somit noch individualisiertere Behandlungskonzepte erstellt werden.

Digitale Operationsmikroskope in der Ohr- und Laterobasischirurgie, Endoskope in der Nasennebenhöhlen- und Frontobasischirurgie sowie Operationsroboter in der zervikalen Weichteil- und Tumorchirurgie bilden die technische Plattform zur Erzeugung mehrdimensionalen Bildmaterials. Zusammen mit der Integration von digitalen radiologischen Befunden bzw. der Implementierung neuartiger optischer Technologien in derartige Operationssysteme könnte KI dem/der Kopf-Hals-Chirurg*in z. B. bei der Orientierung im Operationsgebiet durch die Schaffung einer „erweiterten Realität“ (AR) oder gar durch eine automatisierte Durchführung einzelner Teilschritte einer Operation wirkungsvoll unterstützen. Dabei könnte z. B. eine verbesserte Abgrenzung maligner Prozesse von gesundem Gewebe die Präzision in der Tumorchirurgie weiter erhöhen und hierdurch onkologische Sicherheit bei gleichzeitigem Funktionserhalt steigern.

Allerdings ist es geboten, den zunehmenden Einsatz von KI in der Medizin wachsam zu begleiten. Den enormen Möglichkeiten dieser neuartigen Technologie stehen auch Risiken und Herausforderungen gegenüber, welche einen flächendeckenden KI-Einsatz in der klinischen Praxis erschweren bzw. bisher über weite Strecken unterbunden haben: Herausforderungen im Bereich des Datenschutzes umfassen insbesondere die Einholung von Einwilligungen zur Datennutzung in KI-Systemen, die fachgerechte Datenanonymisierung oder, falls eine Anonymisierung nicht möglich ist, die DSGVO-gerechte Speicherung, Nutzung(sbeschränkung), Löschung und Vertraulichkeit der Daten [8]. Weiterhin bergen unzureichend validierte oder technisch fehlerhafte Algorithmen Gefahren. Ein aktuelles Beispiel hierfür ist die fälschlicherweise durchgeführte Priorisierung des nichtexponierten Fachpersonals für die COVID-19-Impfung an der Universitätsklinik Stanford (Kalifornien, USA) durch einen fehlerhaften KI-Algorithmus [14]. Derartige Zwischenfälle rücken Fragen nach rechtlicher und moralischer Verantwortlichkeit in den Fokus: Fehlerhafte KI-Entscheidungen, insbesondere im Bereich Diagnostik und Therapieplanung, könnten ernsthafte, mitunter lebensbedrohliche Folgen für unsere Patient*innen haben. Aktuell ist nicht pauschal zu beantworten, wer in solchen Fällen haftet – der KI-Entwickler, die Ärzt*innen als Anwender oder aufgeklärte Patient*innen selbst? Insofern ist es unabdingbar, dass Transparenz, Nachvollziehbarkeit und Überprüfbarkeit von KI-gestützten medizinischen Entscheidungen von uns Ärzt*innen engmaschig überwacht werden. Gerade in diesem Bereich lassen sich jedoch beträchtliche Herausforderungen ausmachen. Da die steigende Leistungsfähigkeit moderner ML-Algorithmen nicht zuletzt durch höhere Modellkomplexität und Nutzung umfangreicher Datengrundlagen erreicht wurde, litt die Nachvollziehbarkeit – eine Problematik, die in der einschlägigen Literatur gerne mit der „Black-Box-Metapher“ umschrieben wird: ein dem Behaviorismus entlehntes Sinnbild für fehlende Einsicht in die „Denkprozesse“ der KI. Zwar existieren zahlreiche Ansätze zur Interpretation dieser „Denkprozesse“ im Sinne einer „explainable AI“; anerkannte Richtlinien und Mindeststandards zur Gewährleistung derartiger Transparenz und Überprüfbarkeit in der Medizin fehlen aber bislang. Nur mit adäquater ärztlicher Supervision wird es möglich sein, diese neuartigen Technologien im Sinne der Patient*innen gewinnbringend einzusetzen.

## Fazit für die Praxis

- Der Einsatz von künstlicher Intelligenz (KI) wird die Digitalisierung der Medizin in den nächsten Jahren weiter voranbringen.
- Das enorme positive Potenzial von KI ist auch mit Herausforderungen, z. B. im Bereich des Datenschutzes (Stichwort „gläserner Patient“) mit der Gefahr von Beeinträchtigung der Privatsphäre, Benachteiligung und Ausgrenzung verbunden.
- Aktuelle Untersuchungen deuten darauf hin, dass sich durch die Anwendung von KI die Aussagekraft herkömmlicher Diagnostik, v. a. im Bereich der Bildgebung, weiter steigern lässt.
- In Zukunft könnte KI in „intelligenten Operationssystemen“ chirurgische Präzision und Patientensicherheit weiter erhöhen.
